# Is dissection the *only* way to learn anatomy? Thoughts from students at a non-dissecting based medical school

**DOI:** 10.1007/s40037-015-0206-8

**Published:** 2015-09-09

**Authors:** Salil B. Patel, Daniel Mauro, James Fenn, Dermot R. Sharkey, Conor Jones

**Affiliations:** Peninsula College of Medicine and Dentistry, Royal Devon and Exeter Hospital, EX2 5WD Exeter, UK

**Keywords:** Undergraduate education, Dissection, Anatomy, Online Learning

## Abstract

Anatomical teaching has been centred around dissection for centuries. Generations of doctors have been initiated into the medical profession by cutting into their first cadavers. With the number of donor cadavers available decreasing and medical student numbers increasing, the emphasis placed on dissection has changed dramatically over the past 15 years. However, a solid appreciation of human anatomy is still a necessary part of understanding pathology and treatments. Therefore in light of these changes we ask, is dissection the *only* option? Or are there other options which students can undertake to develop anatomical knowledge?

At Peninsula College of Medicine and Dentistry, no aspect of anatomical teaching involves dissection. We instead utilize medical imaging, life models, pathology lectures and radiologist-lead sessions to teach anatomy in the context of clinical scenarios. As most interactions with patients involve surface anatomy, the rationale behind these teaching sessions is to learn from the ‘outside inwards’. Studies attempting to prove the superiority of one method over another are usually inconclusive [1]. In this article we discuss some of the methods that we as students, at a non-dissection based medical school, have found useful in order to ‘fill the gap’.

Arguments against dissection pivot on both the practical demands of obtaining cadavers and topographical learning outweighing the intricacies of inner structures. However, a study undertaken at the University of Sydney (whereby dissection is not part of anatomy teaching) assigned 29 students to a 34-day full body dissection course [2]. Surgeons and anatomists tasked students with identifying structures at regular intervals throughout and up to one month after the course. The outcome of this study indicated that students’ anatomical knowledge improved significantly on completing the course. The authors suggest that dissection should remain an integral component of anatomical teaching. From our experience, students at Peninsula are equally likely to apply for a similar dissection course to improve anatomical learning. Additionally a study at the University of Melbourne, where dissection is not utilized, illustrated a similar point. First and second year medical students from this university unanimously agreed that dissection would be beneficial [3].

In today’s world of digital technology, students have greater access to educational resources. In a 2012 study, 91 second year medical students were asked if they used internet resources as a source of anatomy teaching [4]. A total of 98 % highlighted YouTube as a source of information and 92 % found anatomy videos beneficial to their anatomy learning. Video sharing platforms including YouTube and other more dedicated anatomy teaching websites such as ‘Funky Professor’, are readily accessible to students from beyond the confines of the anatomy lab. These videos utilize anatomical footage of cadaveric specimens and live patients in surgery in conjunction with medical imaging, plastic models and diagrams to maximize understanding of 3D structures through the 2D medium of video. Although video cannot provide tactile information on tissue textures and lacks the same kinesthetic aspects of learning through dissection, it has the advantage of personalization to the learner. However, kinesthetic experiences may assist students in future endeavours whether during early surgical training or when learning the range of practical tasks undertaken by foundation doctors. Nevertheless, video resources have an array of benefits including allowing students to control the depth of knowledge provided and the style in which it is presented to them: from simple hand-drawn diagrams to an elaborate, layered 3D fly-through. In addition, students can even choose to be taught by experts in the field, and all at a pace that suits them; pausing, rewinding, and revisiting topics as required.

Although criticized for lacking the variability and intricacies of the human body, we have found virtual models a useful adjunct to other methods of learning. These models allow students to isolate structures that may be difficult to appreciate from books or video, by zooming, rotating and even transecting them in order to appreciate anatomical form and relationships. A criticism of dissection surrounds its one directional nature. Once cut, damage to structures remains irreversible. Hence, one could argue that dissection may result in students missing out on learning opportunities due to the lack of flexibility and finite chances. This is of course not an issue for virtual models, where structures can be stripped away or made translucent to follow the course of a nerve or blood vessel, and then reconstructed at the touch of a button. Nevertheless, even plastic life-like models, which medical schools utilize, still do not provide the true physical textures of human anatomy.

Once a basic understanding is obtained, during pre-clinical years, we have found that surgeons in theatre are often keen to teach anatomy during operations. Surgery allows students to apply what they have learnt from books, videos and models, to real-life patients. It is an opportunity to understand the true architecture of the human body, appreciate its complexities and realize the natural variation seen between individuals. However disadvantages include: limited access to theatres (particularly for pre-clinical students), variability in teaching quality, and limited opportunity to explore beyond the scope of the area being operated, as this would inevitably result in unnecessary damage to healthy tissues.

The most important factor is to consider whether doctors who studied at non-dissection universities feel that their anatomical knowledge is sufficient and does not impact on their ability to work as general practitioners and specialists. Therefore as dissection becomes less common throughout UK medical schools, it is essential to reflect on abilities in the years to come. Studies would be beneficial when identifying areas in which current non-dissection universities anatomical curricula can change. In addition, if graduates felt at a disadvantage during foundation years or early core training, such feedback could generate the introduction of classes to help correct a lack of knowledge.

Despite concerns from older generations of doctors, the consequence of removing dissection from the medical curricula does not mean students are left to fend for themselves when learning anatomy. In fact, a multitude of methods are available to bridge gaps left by this tradition. However, studies and first-hand evidence highlight students’ keenness to augment their knowledge by utilizing methods involving cadaveric specimens. Additionally, retrospective studies are essential to help gather first-hand evidence of the benefits and shortfalls in non-dissection teaching.

## Funding

None
